# Protective Effect of Que Zui Tea on d-Galactose-Induced Oxidative Stress Damage in Mice via Regulating SIRT1/Nrf2 Signaling Pathway

**DOI:** 10.3390/molecules29061384

**Published:** 2024-03-20

**Authors:** Yongchao Wang, Yongpeng Wang, Tianrui Zhao, Mengcheng Li, Yudan Wang, Jianxin Cao, Yaping Liu, Zhengxuan Wang, Guiguang Cheng

**Affiliations:** 1 Faculty of Food Science and Engineering, Kunming University of Science and Technology, Kunming 650500, Chinaliuyaping@kust.edu.cn (Y.L.); 2 National and Local Joint Engineering Research Center for Green Preparation Technology of Biobased Materials, Yunnan Minzu University, Kunming 650500, China

**Keywords:** Que Zui tea, oxidative stress, apoptosis, SIRT1/Nrf2 signaling pathway

## Abstract

Que Zui tea (QT) is an important herbal tea in the diet of the ‘Yi’ people, an ethnic group in China, and it has shown significant antioxidant, anti-inflammatory, and hepatoprotective effects in vitro. This study aims to explore the protective effects of the aqueous-ethanol extract (QE) taken from QT against ᴅ-galactose (ᴅ-gal)-induced oxidative stress damage in mice and its potential mechanisms. QE was identified as UHPLC-HRMS/MS for its chemical composition and possible bioactive substances. Thus, QE is rich in phenolic and flavonoid compounds. Twelve compounds were identified, the main components of which were chlorogenic acid, quinic acid, and 6′-*O*-caffeoylarbutin. Histopathological and biochemical analysis revealed that QE significantly alleviated brain, liver, and kidney damage in ᴅ-gal-treated mice. Moreover, QE remarkably attenuated oxidative stress by activating the Nrf2/HO-1 pathway to increase the expression of antioxidant indexes, including GSH, GSH-Px, CAT, SOD, and T-AOC. In addition, QE administration could inhibit the IL-1β and IL-6 levels, which suppress the inflammatory response. QE could noticeably alleviate apoptosis by inhibiting the expressions of Caspase-3 and Bax proteins in the brains, livers, and kidneys of mice. The anti-apoptosis mechanism may be related to the upregulation of the SIRT1 protein and the downregulation of the p53 protein induced by QE in the brain, liver, and kidney tissues of mice. Molecular docking analysis demonstrated that the main components of QE, 6′-*O*-caffeoylarbutin, chlorogenic acid, quinic acid, and robustaside A, had good binding ability with Nrf2 and SIRT1 proteins. The present study indicated that QE could alleviate ᴅ-gal-induced brain, liver and kidney damage in mice by inhibiting the oxidative stress and cell apoptosis; additionally, the potential mechanism may be associated with the SIRT1/Nrf2 signaling pathway.

## 1. Introduction

The dysfunction of organs, including the brain, heart, liver, and kidneys, is closely related to the occurrence of cardiovascular disease, obesity, neurodegeneration, diabetes, and cancer [1]. The liver is an important synthetic and metabolic organ in the body which removes toxic and harmful substances [2]. Currently, liver damage has become a global health problem, causing a 30% mortality rate [3]. As an organ of oxygen consumption, the brain is vulnerable to free radical attacks, including oxygen, nitrogen, or metal radicals, resulting in Alzheimer’s disease, neurological disorders, chronic stress, and other related diseases [4]. The kidney is an important excretory organ that is responsible for the excretion of metabolic waste products, or toxins, in order to maintain a stable internal environment in the body [5]. Therefore, the prevention or minimization of liver, brain, and kidney injuries is important to human health. 

D-galactose (ᴅ-gal), a reducing aldohexose sugar, is an ingredient in many foods, including butter, cheese, milk, yogurt, and honey [6]. Its metabolic products, aldose and hydroperoxide, could lead to the excessive production of reactive oxygen species (ROS) and eventually cause oxidative stress [7,8]. The excessive consumption of ᴅ-gal will result in oxidative damage to vital organs (brain, liver, kidney, etc.) in the body. A D-gal-induced mouse model has been widely used to examine various bioactive agents and explore their possible protective mechanisms.

Many studies have revealed that oxidative stress and inflammation are the main factors responsible for D-gal-induced organ damage [9]. Oxidative stress induced by ROS is an important contributor to many chronic diseases, including metabolic diseases, cognitive disorders, glucose and lipid metabolism disorders, and so on [10]. The excessive production of ROS will attack biomacromolecules, such as protein, lipids, or DNA, resulting in oxidative stress damage [11]. In addition, ROS can activate the nuclear factor-kappa B (NF-κB) signal pathway to promote the expression of inflammatory cytokines, eventually causing an inflammatory response [12]. Without effective interventions or treatments, the damages triggered by oxidative stress and inflammation will induce the cell damage and apoptosis, or even organ damage, thereby accelerating liver- and brain-related diseases [13].

In recent decades, an increasing number of studies have demonstrated that the consumption of exogenous antioxidants or the enhancement of endogenous antioxidant enzyme systems can effectively mitigate oxidative stress damage [14,15]. Edible fruits, vegetables, flowers, and teas contain an abundance of phenolic compounds (i.e., EGCG, caffeic acid, chlorogenic acid, arbutin, and their derivatives), which produce significant anti-oxidative activities [16,17,18,19]. Furthermore, their long-term intake is an effective way to decrease the accumulation of ROS, including hydroxyl radicals, superoxide anion radicals, hydrogen peroxide, etc., and inhibit oxidative stress [15,20]. Some herbal teas, such as snow tea [21], green tea [22], insect tea [23], small-leaved Kuding tea [24], and white tip silver needle [25], were reported to alleviate ᴅ-gal-induced problems in mice by activating the nuclear factor E2-related factor 2 (Nrf2) pathway, thus increasing the expression of antioxidant enzymes and decreasing ROS production [6,7,8,26]. It has been reported that Pu’er tea has an excellent antioxidant ability due to its theaflavins, which have diversity structures of 9-OH groups to scavenge free radicals, providing a basis for the structure–activity relationship of antioxidant activity in tea [27]. Thus, the examination of natural antioxidant agents is a research hotspot regarding human health.

Que Zui tea (QT), an ethnic herbal tea in China, is processed using the tender leaves and buds of *Vaccinium dunalianum* Wight. Long-term consumption of Que Zui tea has beneficial effects on lipid levels, cholesterol levels, vascular occlusion, hypertension, neurasthenic syndrome, and so on [28,29]. It has been reported that QT possesses abundant phenolic compounds, especially 6′-*O*-caffeoylarbutin [30]. According to our previous research, 6′-*O*-Caffeoylarbutin from Que Zui tea ameliorates acetaminophen-induced liver injury via enhancing antioxidant ability and regulating the PI3K signaling pathway [31]. Moreover, the protective effects of QT extracts in acetaminophen-treated mice included the inhibition of oxidative stress, inflammation response, and apoptosis [32,33]. However, the protective effects and potential mechanisms of QT extracts on the brain, liver, and kidney injuries in ᴅ-gal-induced mice were not reported. This study is the first to investigate the protective effects of QE on brain, liver, and kidney damage in ᴅ-gal-treated mice and explore its potential mechanism in term of oxidative stress, inflammation, and cell apoptosis.

## 2. Results

### 2.1. Characterization of QE (the Aqueous-Ethanol Extract of Que Zui Tea) Compounds

**Figure 1:**
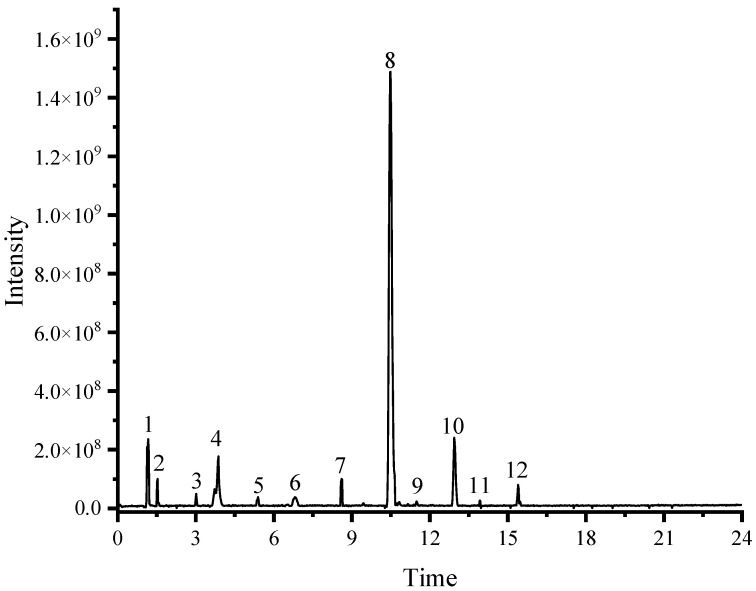
The total ion current chromatograms of ethanol extracts of Que Zui tea (QT) in the negative mode.

In this study, UHPLC-HRMS/MS was used to qualitatively analyze the chemical composition of QE (the aqueous-ethanol extract of Que Zui tea). Table 1 summarizes the details of the identified compounds in QE, including the molecular formula, retention time, mass, and MS/MS fragments. The total ion chromatograph of QE is shown in Figure 1. A total of 12 compounds were identified, including one organic acid (**1**), two arbutin derivatives (**2** and **8**), phenolic acids (**3**, **4**, **5**, **10** and **12**), and four flavonoids (**6**, **7**, **9** and **11**).

### 2.2. Effect of QE on the Body Weight and Organ Indexes

The body weight, food intake, and organ index are very important physiological indicators for the evaluation of overall health and physical development of mice. As shown in Table 2, body weight and food intake were not significantly different in any group (*p* > 0.05). Meanwhile, after injection of the ᴅ-gal for 10 weeks, these organ indexes were prominently reduced in the brains, livers, and kidneys of the Model group, in contrast to the Control group (*p* < 0.01). Interestingly, the QE treatment at doses of 200 and 600 mg/kg evidently elevated the brain (*p* < 0.01), liver (*p* < 0.01), and kidney (*p* < 0.01) indexes in comparison with the Model group.

### 2.3. Effect of QE Treatment on the Organ Damages in ᴅ-Gal-Induced Mice

To evaluate the protective effects of QE on the age-related damage in the brains, livers, and kidneys of the ᴅ-gal-exposed mice, H&E staining was performed, and the function biomarkers related to the brain, liver, and kidneys, such as AChE, AST, ALT and BUN, were also determined in this study.

#### 2.3.1. Protective Effect of QE Treatment on the Brains of ᴅ-Gal-Induced Mice

**Figure 2:**
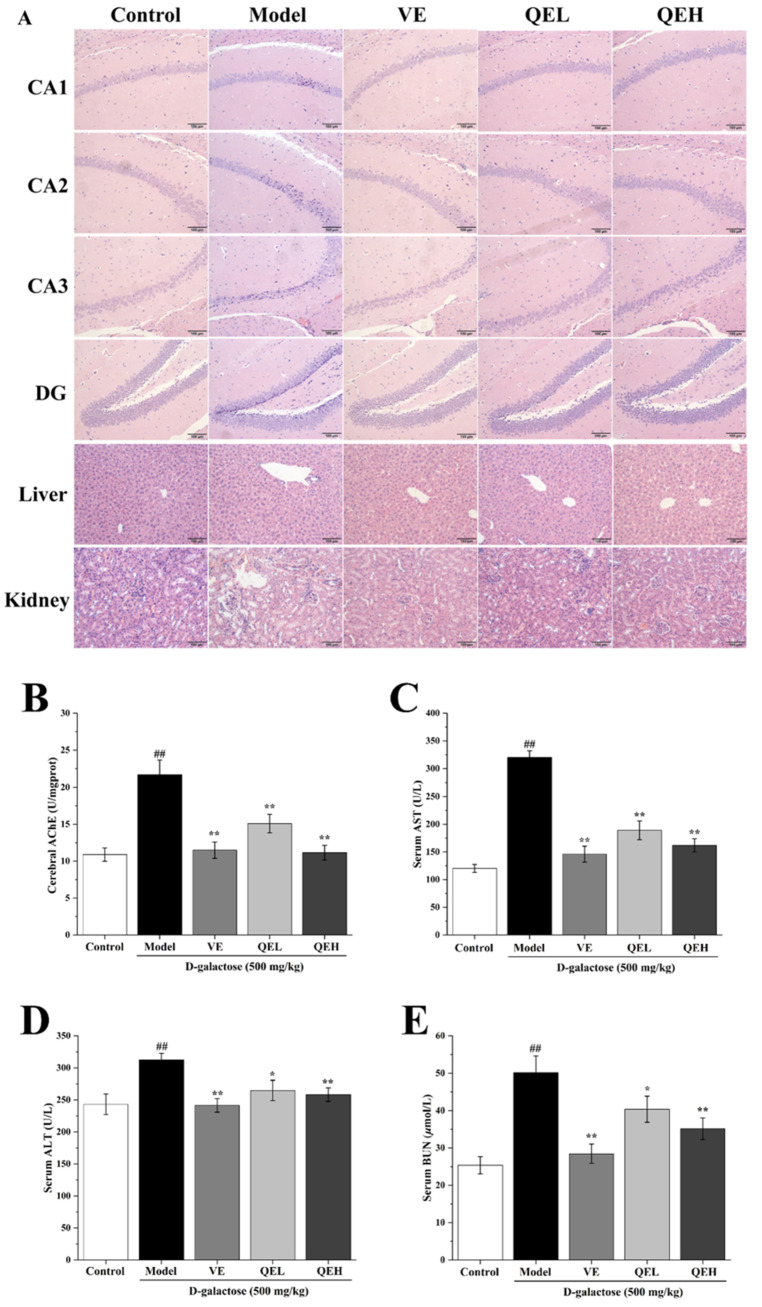
QE relieved age-related brain, liver, and kidney injury in ᴅ-gal-induced mice. Effects of QE on brain, liver, and kidney histopathology changes ((**A**), 200×, scale bars = 100 μm), as well as the activities of cerebral AChE (**B**), serum AST (**C**), ALT (**D**), and BUN (**E**) in ᴅ-gal-treated mice. Values are presented as the mean ± SD (n = 10). ^##^ *p* < 0.01 vs. Control group; * *p* < 0.05, ** *p* < 0.01 vs. Model group. QEL: 200 mg/kg of the aqueous-ethanol extract of Que Zui tea; QEH: 600 mg/kg of the aqueous-ethanol extract of Que Zui tea; ᴅ-gal: ᴅ-galactose; VE: Vitamin E; CA1: cornu ammonis 1; CA2: cornu ammonis 2; CA3: cornu ammonis 3; DG: dentate gyrus; AChE: acetylcholinesterase; AST: Aspartate aminotransferase; ALT: alanine aminotransferase; BUN: blood urea nitrogen.

The hippocampus is the most important part of the brain and consists of cornu ammonis 1 (CA1), cornu ammonis 2 (CA2), cornu ammonis 3 (CA3), and dentate gyrus (DG) regions, all of which consolidate information from short-term to long-term memory [34]. As illustrated in Figure 2A, pyramidal neurons in the CA1, CA2, CA3, and DG regions of the brains in the Control group presented a tight arrangement with a circular and intact nucleus. However, ᴅ-gal administration caused serious neuropathological changes, including many damaged, shriveled, and absent pyramidal neurons, compared to the Control group (Figure 2A). In contrast, the morphological lesions of pyramidal neurons in the CA1, CA2, CA3, and DG regions were markedly improved by the supplementation of QE (Figure 2A). Furthermore, the protective effect of QE at a high dose (600 mg/kg) was similar to that of the VE group. As illustrated in Figure 2B, compared with the Control group, the activity of AChE in the brain was dramatically enhanced by chronic injection of ᴅ-gal (*p* < 0.01), but effectively suppressed by the QE treatment (all *p* < 0.01), which attained the same therapeutic effect as VE treatment (*p* < 0.01).

#### 2.3.2. QE Mitigated Age-Related Liver Injury Induced by ᴅ-Gal

As shown in Figure 2A, in comparison with the Control group, chronic injection of ᴅ-gal caused severe pathohistological alterations in the liver, characterized by the disordered arrangement of hepatocytes, binucleation of hepatocytes, karyopyknosis, and hypertrophy of the nucleus. As expected, the hepatic pathological changes were significantly reversed by the intervention of QE (200 mg/kg), in which only minor histopathological changes were observed in the QEL group. It is worth noting that the hepatoprotective effect in the QEH group was close to the VE and Control group. These observations were supported by the results of liver biomarkers that QEL and QEH treatment significantly decreased the high plasma activities of ALT (Figure 2C, both *p* < 0.01) and AST (Figure 2D, *p* < 0.05 for QEL and *p* < 0.01 for QEH) in D-gal-treated mice, which was similar to the VE group (both *p* < 0.01, Figure 2C,D).

#### 2.3.3. QE Treatment Ameliorated ᴅ-Gal-Elicited Kidney Damage

As found in Figure 2A, normal renal morphological structure was observed in the Control group. Inversely, ᴅ-gal injection generated an extremely damaged renal morphological structure. However, these pathological alterations were conspicuously attenuated by the QE administration. Moreover, the level of plasma blood urea nitrogen (BUN) was dramatically elevated in ᴅ-gal-treated mice compared with the Control group (*p* < 0.01, Figure 2E). Satisfyingly, after the administrations of QE (200 and 600 mg/kg), the BUN contents in the serum were significantly reduced (*p* < 0.05 for QEL, *p* < 0.01 for QEH, Figure 2E).

### 2.4. QE Increased the Antioxidant Abilities of Brain, Liver, and Kidneys in ᴅ-Gal-Treated Mice

**Figure 3:**
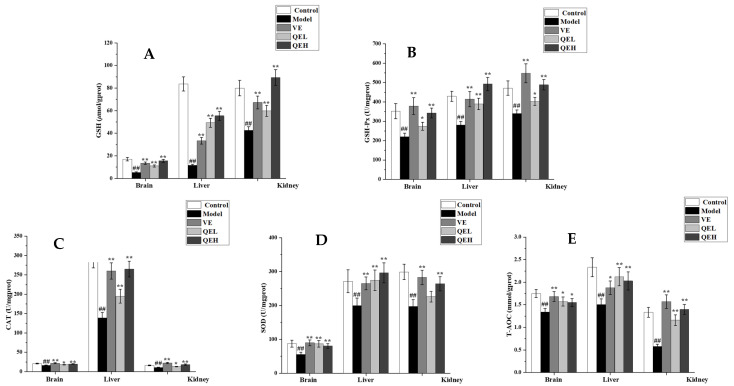
QE renewed the antioxidant ability of the brains, livers, and kidneys of aging mice inhibited by ᴅ-gal. Effects of QE on GSH (**A**), GSH-Px (**B**), CAT (**C**), SOD (**D**), and T-AOC (**E**) contents in the brains, livers, and kidneys of ᴅ-gal-treated mice. Values are presented as the mean ± SD (n = 10). ^##^ *p* < 0.01 vs. Control group; * *p* < 0.05, ** *p* < 0.01 vs. Model group. QEL: 200 mg/kg of the aqueous-ethanol extract of Que Zui tea; QEH: 600 mg/kg of the aqueous-ethanol extract of Que Zui tea; VE: Vitamin E; GSH: glutathione peroxidase; GSH-Px: glutathione peroxidase; SOD: superoxide dismutase; CAT: catalase; T-AOC: total antioxidant capacity.

Oxidative stress is an obvious feature in the process of damage [13]. Thus, an investigation of the effect of QE on the endogenous abilities of antioxidant enzymes was performed in this study. As shown in Figure 3, in comparison with the Control group, the levels of glutathione peroxidase (GSH) and the activities of glutathione peroxidase (GSH-Px), Superoxide Dismutase (SOD), catalase (CAT), and total antioxidant capacity (T-AOC) were significantly decreased in the brains, livers, and kidneys of the mice in the Model group (all *p* < 0.01). However, 200 mg/kg QE remarkably enhanced the activities of GSH-Px (*p* < 0.05, *p* < 0.01 and *p* < 0.05), SOD (*p* < 0.01 and *p* < 0.01), CAT (*p* < 0.05, *p* < 0.01 and *p* < 0.05), T-AOC (*p* < 0.05, *p* < 0.01 and *p* < 0.01), and the contents of GSH (all *p* < 0.01) in the brain, liver, and kidneys compared to those of the Model group (Figure 3). In the kidney of the QEL group, SOD activities in the kidney of the QEL group presented in an increasing trend, but the difference did not attain a statistical significance (*p* > 0.05, Figure 3). Meanwhile, the QEH group indicated higher enzymatic (GSH-Px, SOD and CAT, all *p* < 0.01) or non-enzymatic antioxidant (GSH and T-AOC, all *p* < 0.01) contents in the brain, liver, and kidneys than the Model group, except for SOD in the brain and T-AOC in the brain and liver, similar to, or better than, the VE group (Figure 3).

### 2.5. QE Dampened the ᴅ-Gal-Caused Inflammatory Response in the Brain, Liver, and Kidneys

**Figure 4:**
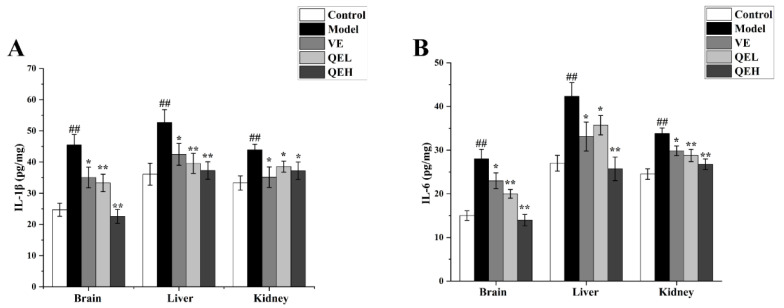
QE alleviated the inflammatory response induced by ᴅ-gal in the brains, livers, and kidneys of mice. The levels of IL-1β (**A**) and IL-6 (**B**) in the brains, livers, and kidneys of aging mice were determined by ELISA kits. Values are presented as the mean ± SD (n = 10). ^##^ *p* < 0.01 vs. Control group; * *p* < 0.05, ** *p* < 0.01 vs. Model group. QEL: 200 mg/kg of the aqueous-ethanol extract of Que Zui tea; QEH: 600 mg/kg of the aqueous-ethanol extract of Que Zui tea; ᴅ-gal: ᴅ-galactose; VE: Vitamin E; IL-6: interleukin 6; IL-1β: interleukin 1 beta.

To evaluate the correlation between QE treatment and inflammation evoked by ᴅ-gal, the production of the inflammatory cytokines interleukin 1 beta (IL-1β) and interleukin 6 (IL-6) in the brain, liver, and kidneys was investigated using an ELISA analysis. As compared to the Control group, prominently higher levels of IL-1β (all *p* < 0.01) and IL-6 (all *p* < 0.01) in the brain, liver, and kidneys were observed in the Model group (Figure 4). Not surprisingly, the QEL and QEH group exhibited prominent inhibition of IL-1β and IL-6 activities in the brain (all *p* < 0.01; all *p* < 0.01), liver (all *p* < 0.01; QEL vs. Model, *p* < 0.05, QEH vs. Model, *p* < 0.01), and kidney (all *p* < 0.05, all *p* < 0.01) compared to that in the Model group, which was similar to that presented in the VE group (Figure 4).

### 2.6. QE Alleviated Apoptosis Induced by ᴅ-Gal in the Brains, Livers, and Kidneys of Mice

**Figure 5:**
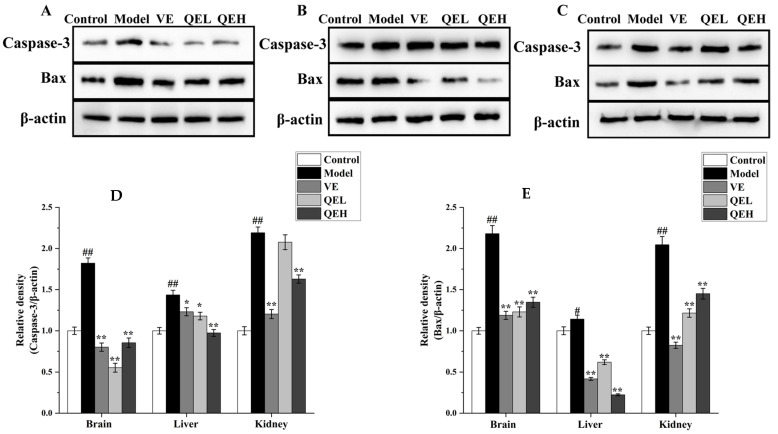
QE mitigated apoptosis induced by ᴅ-gal in the brains, livers, and kidneys of ageing mice. Effects of QE on expressions of Caspase-9 and Bax in the brains (**A**), livers (**B**), and kidneys (**C**) of ᴅ-gal-treated mice. Quantification of Caspase-9/β-actin ratio (**D**) and Bax/β-actin ratio (**E**) in the brain, liver, and kidneys. Values are presented as the mean ± SD (n = 5). ^#^ *p* < 0.05, ^##^ *p* < 0.01 vs. Control group; * *p* < 0.05, ** *p* < 0.01 vs. Model group. QEL: 200 mg/kg of the aqueous-ethanol extract of Que Zui tea; QEH: 600 mg/kg of the aqueous-ethanol extract of Que Zui tea; ᴅ-gal: ᴅ-galactose; VE: Vitamin E; Caspase-3: cysteinyl aspartate specific proteinase-3; Bax: Bcl-2-associated X protein.

Excessive apoptosis of cells can lead to irreversible tissue damage. Consequently, Western blotting was used to detect the effect of QE on the expression of cysteinyl aspartate specific proteinase-3 (Caspase-3) and Bcl-2-associated X protein (Bax) in the brains, livers, and kidneys of ᴅ-gal-treated mice. As illustrated in Figure 5, chronic exposure to ᴅ-gal significantly strengthened Caspase-3 (all *p* < 0.01) and Bax (*p* < 0.01 for brain, *p* < 0.05 for liver, and *p* < 0.01 for kidney) protein expression in the brain, liver, and kidneys relative to the Control group. On the contrary, treatment of QE at a dose of 200 mg/kg prominently minimized the expression levels of Caspase-3 (*p* < 0.05 for brain and *p* < 0.01 for liver) and Bax (all *p* < 0.01) protein compared to the Model group, but no significant difference was presented in the kidneys of the QEL groups (*p* > 0.05, Figure 5). Moreover, similar results were also observed in the QEH group (all *p* < 0.01), and the inhibition of 600 mg/kg QE treatment on Caspase-3 and Bax protein in the brains and livers of mice treated with ᴅ-gal was better than or close to the VE group (Figure 5).

### 2.7. QE Relieved Oxidative Stress via Modulating the Nrf2 Signaling Pathway in ᴅ-Gal-Treated Mice

**Figure 6:**
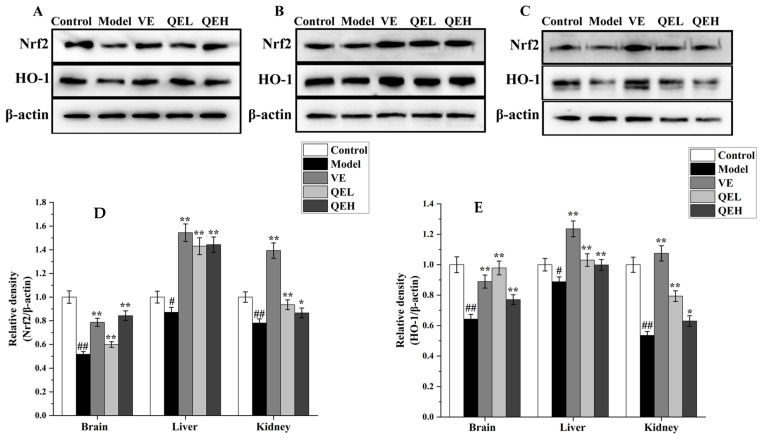
QE treatment counteracted oxidative stress in the brains, livers, and kidneys of ᴅ-gal-exposed mice by regulating Nrf2 signaling pathway. Effects of QE on expressions of Nrf2 and HO-1 in the brains (**A**), livers (**B**), and kidneys (**C**) of aging mice. Quantification of Nrf2/β-actin ratio (**D**) and HO-1/β-actin ratio (**E**) in the brain, liver, and kidneys. Values are presented as the mean ± SD (n = 5). ^#^ *p* < 0.05, ^##^ *p* < 0.01 vs. Control group; * *p* < 0.05, ** *p* < 0.01 vs. Model group. QEL: 200 mg/kg of the aqueous-ethanol extract of Que Zui tea; QEH: 600 mg/kg of the aqueous-ethanol extract of Que Zui tea; ᴅ-gal: ᴅ-galactose; VE: Vitamin E; Nrf2: nuclear factor-erythroid 2-related factor 2; HO-1: Heme Oxygenase-1.

To further explore the molecular mechanism of QE that enhances the antioxidant capacity of the brain, liver and kidneys, Western blot analysis was processed to evaluate the expression levels of nuclear factor-erythroid 2-related factor 2 (Nrf2) and Heme oxygenase-1 (HO-1) protein. As listed in Figure 6, ᴅ-gal administration prominently declined the proportion of Nrf2/β-actin (*p* < 0.05 or *p* < 0.01) and HO-1/β-actin (*p* < 0.05 or *p* < 0.01) in the brains, livers, and kidneys of the Model mice as compared with the Control group. Conversely, the treatment with QE (200 and 600 mg/kg) markedly elevated the expression levels of Nrf2 (*p* < 0.05 or *p* < 0.01) and HO-1 (*p* < 0.05 or *p* < 0.01) in the brain, liver, and kidneys compared to those of the Model group (Figure 6); particularly, the Nrf2 and HO-1 protein expression in the brain and liver were close to those in the VE group.

### 2.8. QE Alleviated D-Gal-Induced Brain, Liver, and Kidney Damages via SIRT1 Signaling Pathway

**Figure 7:**
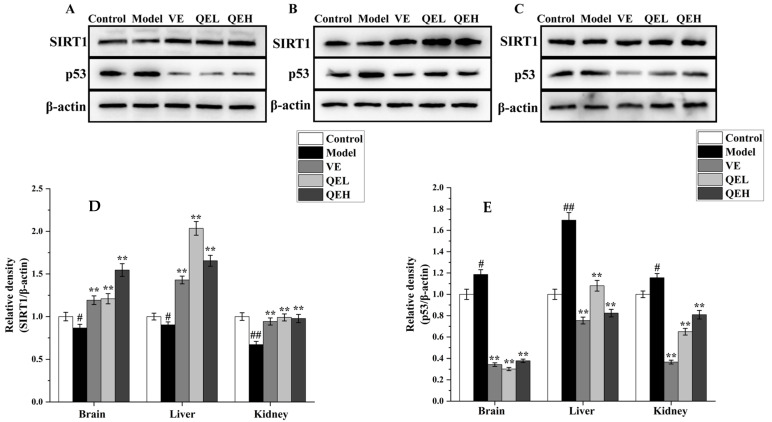
Effects of QE treatment on the expression level of the SIRT1 signaling pathway in the brains, livers, and kidneys of ᴅ-gal-treated mice. Western blots of SIRT1 and p53 in the brains (**A**), livers (**B**), and kidneys (**C**) of ᴅ-gal-induced aging mice. Quantification of SIRT1/β-actin ratio (**D**) and p53/β-actin ratio (**E**) in the brain, liver, and kidneys. Values are presented as the mean ± SD (n = 5). ^#^ *p* < 0.05, ^##^ *p* < 0.01 vs. Control group; ** *p* < 0.01 vs. Model group. QEL: 200 mg/kg of the aqueous-ethanol extract of Que Zui tea; QEH: 600 mg/kg of the aqueous-ethanol extract of Que Zui tea; ᴅ-gal: ᴅ-galactose; VE: Vitamin E; SIRT1: silent information regulator 1; p53: tumor suppressor gene.

To explore the potential mechanism of QE for alleviating organ damage, the SIRT1/p53 signaling pathway protein was determined in the brains, livers, and kidneys of mice using Western blot analysis. As illustrated in Figure 7, we noticed a markedly reduced ratio of SIRT1/β-actin and a dramatically promoted ratio of p53/β-actin in the brains, livers, and kidneys of the mice, which was in contrast to the Control group (*p* < 0.05 or *p* < 0.01, Figure 7). However, QE treatment at dosages of 200 and 600 mg/kg could conspicuously upregulate the expression levels of the SIRT1 protein (*p* < 0.05 or *p* < 0.01) and downregulate the expression levels of the p53 protein (*p* < 0.05 or *p* < 0.01) in the brains, livers, and kidneys of ᴅ-gal-treated mice in comparison with the Model group (Figure 7).

### 2.9. Molecular Docking Results of Principal Components on Nrf2 and SIRT1

**Figure 8:**
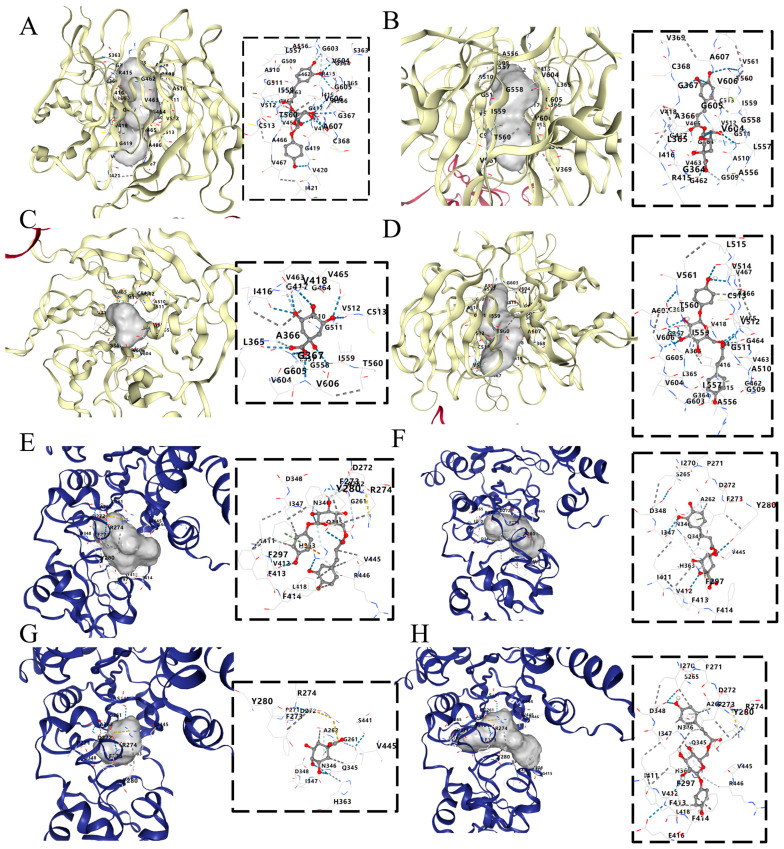
Molecular docking between principal components and Nrf2: 6′-*O*-caffeoylarbutin (**A**); chlorogenic acid (**B**); quinic acid (**C**); robustaside A (**D**); SIRT1: 6′-*O*-caffeoylarbutin (**E**); chlorogenic acid (**F**); quinic acid (**G**); robustaside A (**H**). Nrf2: nuclear factor-erythroid 2-related factor 2; SIRT1: silent information regulator 1.

The extent of the major chemical components in QT that bind to key target proteins was assessed by the molecular docking method. Molecular docking conformations were analyzed and visualized by CB-Dock2. Binding Energy (BE) represents the binding energy between the ligand and receptor. The chain break threshold is 7. The maximum distance value of the Strong H-bond is 3.4, the maxiumum dist value of the salt bridge is 4, and the maximum dist value of the Weak H-bond is 3.8. The maximum dist value of the acceptor–acceptor is 3, the minimum degree value of the hydrogen bond is 90, and the maximum deg value of the hydrogen bond is 180. The results of molecular docking showed that the main binding forces were hydrogen bonds and van der Waals forces. As shown in Figure 8, 6′-*O*-caffeoylarbutin (BE = −10.4), chlorogenic acid (BE = −9.4), quinic acid (BE = −7.2), and Robustaside A (BE = −10.4) showed strong binding ability to Nrf2, and, as shown in Figure 7, 6′-*O*-caffeoylarbutin (BE = −9.1), chlorogenic acid (BE = −8.6), quinic acid (BE = −6.1), and Robustaside A (BE = −9.8) had a strong binding capacity for SIRT1. Table 3 summarizes the detailed molecular docking information of the chemical components and key target proteins.

## 3. Discussion

Multiple system organ failures, including chronic liver and kidney damage, have become a global health concern [35]. Therefore, methods that can be used to alleviate the health problems caused by the failure of these organs have attracted the attention of countries around the world. In general, the processes of organ damage are characterized by excessive accumulation of ROS-inducing oxidative stress, excessive inflammatory cytokine levels (causing an inflammatory response), and apoptosis. Hence, natural products with good antioxidant capacity, anti-inflammatory effects, and low toxicity have become important resources for preventing organ damage. Qui Zui tea (QT) is an ethnic herbal tea which has been reported to have antioxidant abilities and anti-inflammation effects in vitro [32,36]. However, whether or not Que Zui tea extract has organ-protecting effects remains unclear. Therefore, this study aimed to elucidate the protective effects of QT and provide a theoretical basis for the further development of human health foods.

The brain is a very important organ of the body, and it is particularly vulnerable to ROS-induced oxidative stress [37]. Studies have revealed that about 20% of the body’s oxygen consumption occurs in the brain, but the brain lacks antioxidants and antioxidative enzymes [38,39]. Therefore, without the supplementation of exogenous antioxidants, the excessive ROS may result in neurological decline or cognitive dysfunction in the brain, such as Alzheimer’s disease, neurodegenerative disease, etc. [40]. Particularly, the damage to the CA1, CA2, CA3, and DG regions in the brain will directly affect the transition of information from short-term to long-term memory. Additionally, the liver and kidneys are the foremost detoxification and metabolic organs, playing a regulatory role in maintaining body health [41]. In particular, the liver is also the most important organ for the production of various antioxidative enzymes and antioxidants, such as SOD, CAT, GSH, etc., which could spread through the blood to other parts of the body, including the brain [42]. Therefore, the health status of the body’s organs is very important. The organ index is an important macro-index used to evaluate the health status of organs [43]. When the organ declines due to damage, the organ’s index will decrease, which is also combined with organ failures [44]. In this study, QE treatment significantly decreases the organ index of the brain, liver, and kidneys, caused by the D-gal treatment, indicating that QE treatment could dramatically relieve the atrophy of the brain, liver, and kidneys in D-gal-treated mice.

It is well known that the activities of ALT and AST are very important biomarkers for liver damage [45]. When liver damage occurs, the hepatic cell membranes will be destroyed, resulting in the immigration of ALT and AST from the liver cells to the serum [46]. Therefore, higher activities of ALT and AST in the serum represent more serious liver damage. As a biomarker for the kidney, a high level of BUN means the glomeruli cannot filter the urine properly, which is one of the characteristics of kidney damage [47]. Similarly, a high AchE activity in the brain will destroy the structure of acetylcholine, a very important neurotransmitter that regulates brain signals, causing cognitive impairment and memory loss [48]. Hence, these biomarkers represent vital indicators for determining the degree of damage in the body’s organs. Many studies have reported that natural products from *Anneslea fragrans*, *Anthurium schlechtendalii Kunth*, Mango, etc. had protective effects on the liver, kidneys, and brain via the regulation of these biomarkers [49,50,51]. Our results also revealed that QE treatment could significantly decrease the plasma activities of ALT and AST, the BUN level in the kidney, and the AchE activity in the brain. Moreover, the histological photomicrographs further verified this result, where QE treatment remarkably alleviated D-gal-induced brain, liver, and kidney damage.

Nrf2 is a very important nuclear transcription factor, and it plays a pivotal regulatory role in maintaining the balance of oxidation and reduction in the body [52]. Normally, the Nrf2 is locked by Kelch-like ECH-associated protein-1 (Keap1) and Cullin3 (Cul3) with no activity in the cytoplasm [53]. After the stimulation of ROS, or oxidative stress productions, the Nrf2 will be released from the complex of Nrf2-Keap1-Culs and will transfer into the nucleus to bind with the antioxidant response element (ARE), eventually promoting the transcription of various antioxidant enzymes (GSH-Px, CAT, SOD and HO-1), GSH synthesis, and metabolism [54]. These antioxidant enzymes play vital roles in scavenging excessive ROS. For example, the O^2−^ can be translated into H_2_O_2_ with the catalyzation of SOD, and the latter will be further resolved to H_2_O and oxygen by CAT catalyzation [55]. Additionally, GSH is the most abundant antioxidant in the body that can directly scavenge free radicals, including lipid and metal radicals, through its unique redox reactions with the catalyzation of GSH-Px and glutathione reductase (GR) [56]. Thereby, the activation of the Nrf2-regulated antioxidant pathway is the key to the evaluation of exogenous compounds that activate the endogenous antioxidant ability. As the main chemical compounds of QE, 6′-*O*-caffeoylarbutin, chlorogenic acid, and quinic acid were reported to have antioxidant ability by activating the Nrf2 pathway [29,57,58]. More precisely, our result regarding molecular docking further verified that these compounds could bind to the Nrf2 protein. In addition, our result further demonstrated that QE could activate the Nrf2 pathway, increase the activities of antioxidant enzymes and TOC, promote GSH synthesis, and reduced ROS accumulation.

Moreover, the inflammatory response is closely related to the oxidative stress. Studies have shown that oxidative stress could induce expressions of inflammatory cytokines, such as IL-6 and IL-1β, which result in an inflammatory response. HO-1 is the rate-limiting enzyme for the degradation of heme into biliveric acid, Fe^2+^, and CO in the body. Currently, accumulated studies reveal that the Nrf2/HO-1 pathway is responsible for the regulation of inflammatory responses by decreasing the levels of inflammatory cytokines in the organs of the brain, liver, kidneys, etc. The same result was also found in our study, which showed that QE markedly increased the HO-1 protein expressions in the brain, liver, and kidneys, and decreased the IL-6 and IL-1β levels. It was worth noting that there were significant negative relationships among HO-1 expression and IL-6 (r = −0.923) or IL-1β (r = −0.916) levels.

**Figure 9:**
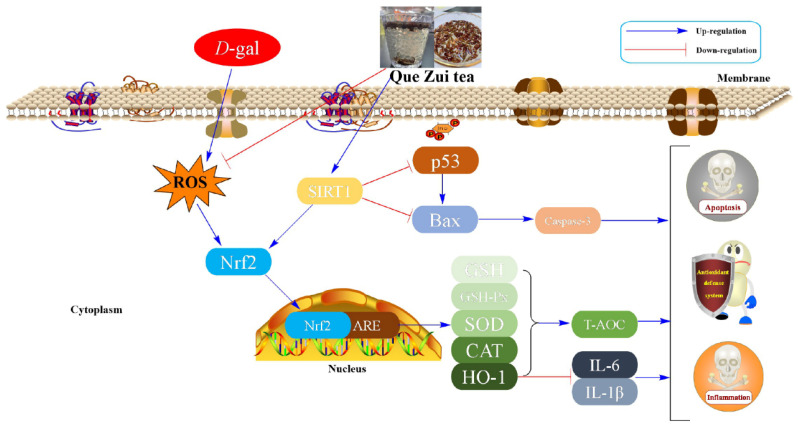
The proposed mechanisms of QE relieving ᴅ-gal-elicited aging in mice. ᴅ-gal: ᴅ-galactose; ROS: reactive oxygen species; Sirt1: silent information regulator transcript-1; p53: tumor protein 53; Bax: Bcl-2 associated X; Caspase-3: Cysteinyl aspartate specific proteinase-3; Nrf2: nuclear factor-erythroid 2-related factor-2; HO-1: Heme oxygenase-1; GSH: glutathione peroxidase; GSH-Px: glutathione peroxidase; SOD: superoxide dismutase; CAT: catalase; T-AOC: total antioxidant capacity; IL-6: interleukin 6; IL-1β: interleukin 1 beta.

The Sirtuins (SIRT1) are a family of histone deacetylases dependent on nicotinamide adenine dinucleotide (NAD+), which plays an important role in organ damage [59]. It has been documented that SIRT1 could deacetylate p53 to improve oxidative stress, apoptosis, and damage [21]. Furthermore, SIRT can not only promote the expression of Nrf2 but also minimize the expression of downstream protein Bax [60]. Numerous studies have demonstrated that apoptosis triggered by oxidative stress plays a vital role in brain, hepatic, and renal injury, appearing in a wide range of senescence-related diseases [61,62]. Convincing evidence has proven that apoptosis makes a critical difference in the damage process caused by ᴅ-gal [63]. According to the previous study, Caspase-3 is considered to be a critical mediator of apoptosis, playing a crucial role in the execution of programmed cell death [64]. Bax is a key gene that promotes apoptosis within the Bcl-2 protein family and is involved in cellular damage. Inhibiting Bax expression has been shown to significantly reduce apoptosis induced by hydrogen peroxide, free radicals, and inflammatory cytokines [65]. Therefore, the SIRT1/Nrf2/p53 pathway has a vital regulatory effect on alleviating D-gal-induced oxidative stress, inflammatory response, and apoptosis (Figure 9). Our results showed that the main chemical compounds (6′-*O*-caffeoylarbutin, chlorogenic acid, quinic acid, robustaside A) of QE could bind well to the protein of SIRT1. Maybe these compounds play regulatory roles in the anti-apoptosis of QE in D-gal-induced aging mice. This hypothesis was supported by other studies in which both chlorogenic acid and quinic acid were reported to have an anti-apoptosis ability in vivo [66,67]. Finally, the Western blot analysis results in this work further demonstrated that QE could regulate the protein expressions of SIRT1 and p53. These observations suggested that the therapeutic effect of QE against age-related brain, liver, and kidney dysfunction was closely related to the activation of the SIRT1 signaling pathway.

## 4. Materials and Methods

### 4.1. Reagents and Plant Material

Formic acid and acetonitrile of LC/MS grade were provided by Merck (Darmstadt, Germany). Ultrapure water was gained using a Milli-Q system (Millipore, Bedford, MA, USA). The RIPA lysis buffer and ᴅ-gal (purity above 99%) were obtained from Sigma-Aldrich (St. Louis, MO, USA). Vitamin E (VE, purity above 97%) was supplied by Shanghai yuanye Bio-Technology Co., Ltd. (Shanghai, China). The 5 × loading buffer and phosphatase and protease inhibitors were obtained from Servicebio (Wuhan, China). The kits for biochemical analysis of the bicinchoninic acid protein (BCA) protein assay, aspartate aminotransferase (AST), alanine aminotransferase (ALT), blood urea nitrogen (BUN), glutathione peroxidase (GSH), glutathione peroxidase (GSH-Px), total anti-oxidation capability (T-AOC), catalase (CAT), superoxide dismutase (SOD), and acetylcholin-esterase (AChE) were obtained by Beyotime Biotechnology (Shanghai, China). ELISA kits specific for mouse interleukin-1β (IL-1β) and interleukin-6 (IL-6) were bought from MultiSciences (Lianke) Biotech (Hangzhou, China). The primary antibodies for Western blot analysis, β-actin (AC026), nuclear factor-erythroid-2-related factor 2 (Nrf2, A0674), heme oxygenase-1 (HO-1, A1346), sirtuin 1 (SIRT1, A11267), tumor suppressor protein 53 (p53, A0263), Caspase-3 (A2156), and Bax (A0207), were supplied by Abclone (Wuhan, China).

The QT was collected in May 2022 from Wuding County, Yunnan Province, China. The Que Zui tea specimen (cheng-20220501-VD) was authenticated by Prof J. X. Cao and stored at the Faculty of Food Science and Engineering, Kunming University of Science and Technology.

### 4.2. Preparation of QE

QE was extracted using the ultrasound-assisted extraction method reported in the previous study [31]. First, the dried QT powder (2 kg) was degreased by petroleum ether (6 L) 3 times and then extracted by 80% aqueous-ethanol solvent at a ratio of 1:3 (*w/v*) associated with an ultrasonic cleaning bath (200 W, 0.5 h per time, Mereal. Sonic, Wuxi, China). After the collection and centrifugation (1500× *g*, 10 min), the supernatant was condensed using a rotary evaporator (Hei-V AP, Heidolph, Schwabach, Germany, 50 °C) to obtain the extract (QE), which was then freeze-dried for further investigation.

### 4.3. Qualitative Analysis of QE Was Performed by UPLC-LC/MS

The extract QE of finch pea tea was dissolved and filtered with mass spectrometric acetonitrile. The analysis was performed using an ultra-high performance liquid chromatography mass spectrometry instrument. The liquid phase conditions were as follows: Agilent Poroshell 120 EC-C18 chromatographic column (2.1 × 100 mm, 1.9 μm), column temperature chamber temperature of 30 °C, injection volume of 2 μL, and flow rate of 0.2 mL/min. The mobile phase consisted of acetonitrile and 0.1% formic acid water, and the gradient elution program was 0–5 min with 10–15% acetonitrile for 5–10 min, 15–20% acetonitrile; 10–20 min, 20–40% acetonitrile; 20–25 min, 40–60% acetonitrile. The other parameters of analysis in mass spectrometer were installed in accordance with our previous work [68].

### 4.4. Animals and Experimental Design

Fifty specific-pathogen-free (SPF) Kunming male mice (8 weeks old, 25−30 g) were purchased by Hunan SJA Laboratory Animal Co., Ltd. (Changsha, China) (certificate number: SCXK (Xiang) 2022–0007). The mice were housed in a standard environment (23 ± 2 °C, 12/12 light-dark cycle, and 40–75% humidity) and provided with a standard chow diet (Beijing Keao Xieli Feed Co., Ltd., Beijing, China) containing 11.85% fats (soybean oil, lard), 23.07% proteins (casein, L-cystine), and 65.08% carbohydrates (corn starch, maltodextrin, sucrose, cellulose), and free access to water. All the experimental animal protocols were in accordance with the Guidelines for the Care and Use of Experimental Animals and authorized by the Animal Experimental Ethics Committee of Kunming University of Science and Technology (Approval number, 202207-01).

A ᴅ-gal-treated mouse model was established in accordance with the previous study [69]. The dosages of QE were selected according to our previous study [70]. The mice were acclimatized for one week and were randomly divided into five groups (n = 10): (1) control group (Control); (2) model group (Model); (3) positive group (VE, 200 mg/kg b.w.); (4) low dose of QE treatment group (QEL, 200 mg/kg b.w.); and (5) high dose of QE treatment group (QEH, 600 mg/kg b.w.). Except for the Control group, all mice received 300 mg/kg ᴅ-gal (dissolved in bacteria-free saline) by subcutaneous injection in the neck once daily for 10 consecutive weeks. The VE and QE were dissolved with distilled water with 0.5% Tween 80 for the intragastric administration. The dosages of QE and ᴅ-gal were selected depending on our preliminary experiments [31,70].

After the animal experiment ended, all mice fasted for 24 h and then were weighed and sacrificed using anesthetic (1% pentobarbital sodium). The blood was collected and then centrifuged at 3000× *g* for 10 min to obtain the serum. Then, the brain, liver, and kidneys were rapidly harvested and weighed in order to calculate the organ index (organ index = organ weight (mg)/body weight (g)). All the sample were stored at 80 °C for further study.

### 4.5. The Investigations of Biochemical Parameters in the Serum and Tissues

The tissues of the brain, liver, and kidneys were separately mixed with 0.9% pre-cooled saline at a solid-to-liquid ratio of 1:9 and then homogenized using Scientz-48L cryogenic high throughput tissue grinder (Xinzhi Biotechnology Co., Ltd., Ningbo, China). Then, the homogenate of the brain, liver, and kidney tissue was centrifuged (3000× *g*, 4 °C) for 10 min to obtain the supernatant for the determinations of biochemical parameters. The corresponding biochemical indicators of AST, ALT, BUN, AChE, GSH, GXH-Px, CAT, SOD, T-AOC (Beyotime Biotechnology, Shanghai, China), IL-1β, and IL-6 (MultiSciences (Lianke) Biotech., Hangzhou, China) in serum or brain, liver, and kidney tissues were determined by the methods described in the commercially available assay kits.

### 4.6. Hematoxylin-Eosin Stains

The method of the Hematoxylin-eosin stain (H&E) was described in our previous study [71]. Some brain, liver, and kidney tissues were fixed in 4% paraformaldehyde. After dehydration with 50–100% ethanol and infiltration with xylene in sequence, the sample was embedded in paraffin, which was then cut into 5 μm slices. These slices of the brain, liver, and kidney were regularly stained with hematoxylin-eosin (H&E). The staining sections were observed using an Olympus IX83 microscope (Tokyo, Japan) fin order to evaluate the histopathological changes.

### 4.7. Western Blotting Analysis

Western blot analysis was performed according to the previous report [31]. The tissues (brain, liver, and kidney) were mixed with the cold-prepared lysis buffer (supplemented with 1 × phosphatase and protease inhibitors) and then homogenized using a Scientz-48 L cryogenic grinder (Xinzhi Biotechnology Co., Ltd., Ningbo, China). After centrifugation, the supernatant was obtained, and its protein concentration was determined by the BCA protein assay kit. The supernatant was added to a 5 × loading buffer (*v/v* = 1:4) and then boiled in water for 10 min to obtain fixed protein. Equal amounts of protein (20 μg) were isolated by SDS-polyacrylamide gel electrophoresis and then transferred onto an NC membrane. After blocking for 1 h with 5% skim milk solution, the NC membrane was incubated overnight with the corresponding primary antibodies of β-actin (AC026, Abclone, Wuhan, China), nuclear factor-erythroid-2-related factor 2 (Nrf2, A0674, Abclone), heme oxygenase-1 (HO-1, A1346, Abclone), sirtuin 1 (SIRT1, A11267, Abclone), tumor suppressor protein 53 (p53, A0263, Abclone), Caspase-3 (A2156, Abclone), and Bax (A0207, Abclone), at 4 °C, and with the secondary antibody (Santa Cruz Biotechnology, Santa Cruz, CA, USA) for 2h at room temperature. The immune-active proteins were visualized using an enhanced chemiluminescent detection reagent (Millipore, Billerica, MA, USA) and determined by a VILBER Fusion FX7 imaging system (Vilber Lourmat, Marne-la-Vallée, France).

### 4.8. Molecular Docking Methodology

CB-Dock2 is a protein-ligand blind docking server [72]. The protein-surface-curvature-based cavity detection approach (Cur Pocket) was performed to guide the molecular docking with Auto Dock Vina (version 1.1.2) [72,73]. The structural data of proteins and ligands were input into the CB-Dock2 server (https://cadd.labshare.cn/cb-dock2/php/index.php accessed on 17 March 2024), and the results were calculated and visualized. These principal components were verified by pairwise docking with signaling pathway proteins. AutoDock Tools (4.2) and Discovery Studio (4.5) software were used to analyze the molecular docking results.

### 4.9. Statistical Analysis

The study data were expressed as mean ± standard deviation (SD). The significant difference was analyzed by one-way ANOVA and Tukey’s test using Origin 8.5 software. Quantification was carried out using image analysis software (ImageJ, 1.46a; NIH, Bethesda, MD, USA). A *p*-value less than 0.05 (*p* < 0.05) was considered as statistically significant.

## 5. Conclusions

In summary, this study provides the first systematic evidence that QE treatment can significantly alleviate ᴅ-gal-induced dysfunction in the brain, liver, and kidneys during the D-gal-induced damage process by increasing antioxidant ability, decreasing IL-1β and IL-6 levels, and suppressing apoptosis. The potential mechanism may be related to the regulation of the SIRT1/Nrf2 signaling pathway. However, further extensive research is needed to investigate the effects of QE on spatial learning and memory impairments induced by ᴅ-gal during the damage process.

## Figures and Tables

**Table 1:** Chemical composition of the ethanolic extract of Que Zui tea was identified using UHPLC-ESI-HR-MS/MS in negative ion mode.

Peak	T_R_ (min)	[M-H]^−^ (*m*/*z*)	MS/MS Fragment Ions	Error (ppm)	Molecular	Compounds
1	1.17	191.0562	85.0284, 93.0336, 191.0557	1.389	C_7_H_12_O_6_	quinic acid
2	1.53	271.0823	71.0124, 108.0202, 110.0317	7.044	C_19_H_12_O_2_	arbutin
3	3.00	341.0877	59.0124, 135.0439, 161.0234, 179.0342, 221.0445	3.024	C_15_H_18_O_9_	6-*O*-trans-caffeoyl-d-glucopyranose
4	3.83	353.0878	85.0280, 127.0389, 191.0553	3.091	C_16_H_18_O_9_	chlorogenic acid
5	5.35	179.0341	89.0382, 134.0361, 135.0448	1.144	C_9_H_8_O_4_	caffeic acid
6	6.86	319.0458	57.0332, 83.0123, 125.0232, 193.0133	3.311	C_15_H_12_O_8_	Ampeloptin
7	8.60	595.1670	161.0233, 323.0770, 433.1137	2.291	C_27_H_32_O_15_	Neoeriocitrin
8	10.49	433.1137	161.0233, 179.0339	1.886	C_21_H_22_O_10_	6′-*O*-caffeoylarbutin
9	11.49	463.1247	139.0389, 161.0233	2.531	C_22_H_23_ClO_11_	Peonidin 3-*O*-glucoside chloride
10	12.96	417.1224	145.0284, 163.0390	2.712	C_21_H_22_O_9_	robustaside A
11	13.92	447.1296	145.0284, 160.0155, 175.0390, 193.0499	2.453	C_21_H_20_O_11_	kaempferol-3-*O*-*β*-d-glucopyranoside
12	15.42	475.1245	161.0233, 179.0340, 475.1274	2.032	C_23_H_24_O_11_	dunalianoside E

T_R_: Retention time.

**Table 2:** Effect of QE on body weight, food intake, and organ coefficient.

	Body Weight (g)		Food Intake (g per Mouse per Day)	Organ Index (mg/g)		
Group	Initial	Final		Brain	Liver	Kidney
Control	36.74 ± 2.42	41.70 ± 2.89	4.64 ± 0.39	9.42 ± 0.13	44.12 ± 1.69	6.71 ± 0.12
Model	37.49 ± 1.61	44.40 ± 0.33	4.79 ± 0.40	8.90 ± 0.15 ^#^	39.71 ± 2.05 ^#^	6.42 ± 0.07 ^#^
VE	38.21 ± 3.56	43.03 ± 1.48	4.33 ± 0.44	9.23 ± 0.14 *	45.18 ± 2.25 *	6.72 ± 0.14 *
QEL	35.62 ±3.60	41.33 ± 2.32	4.65 ± 0.42	9.15 ± 0.10 *	43.13 ± 1.25 *	6.83 ± 0.18 *
QEH	39.15 ±2.12	44.79 ± 0.95	4.59 ± 0.38	9.11 ± 0.13 *	46.05 ± 1.41 *	6.80 ± 0.21 *

Values are presented as the mean ± SD (n = 10). ^#^ *p* < 0.05 vs. Control group; * *p* < 0.05 vs. Model group. QEL: 200 mg/kg of the aqueous-ethanol extract of Que Zui tea; QEH: 600 mg/kg of the aqueous-ethanol extract of Que Zui tea; ᴅ-gal: ᴅ-galactose; VE: Vitamin E.

**Table 3:** Molecular docking parameters of Nrf2 and SIRT1.

	BE (Kcal/mol)	Cavity Volume (Å^3^)	Center (x, y, z)	Docking Size (x, y, z)	Chain Break Threshold
Nrf2 (7k2a)					
6′-*O*-caffeoylarbutin	−10.4	536	−28, 19, 6	26, 26, 26	7
Chlorogenic acid	−9.4	536	−28, 19, 6	23, 23, 23	7
Quinic acid	−7.2	536	−28, 19, 6	25, 17, 28	7
Robustaside A	−10.4	536	−28, 19, 6	23, 23, 23	7
SIRT1 (4zzi)					
6′-*O*-caffeoylarbutin	−9.1	6561	7, 47, −4	23, 35, 23	7
Chlorogenic acid	−8.6	6561	7, 47, −4	23, 35, 23	7
Quinic acid	−6.1	6561	7, 47, −4	26, 35, 28	7
Robustaside A	−9.8	6561	7, 47, −4	23, 35, 23	7

## Data Availability

The data presented in this study are available on request from the corresponding author.
